# Development of a course based on BEAM robots to enhance STEM learning in electrical, electronic, and mechanical domains

**DOI:** 10.1186/s41239-021-00311-9

**Published:** 2022-02-03

**Authors:** Carlos Boya-Lara, Doris Saavedra, Aaron Fehrenbach, Angel Marquez-Araque

**Affiliations:** 1 Universidad Interamericana de Panamá, 07095 Panama City, Panama; 2 Grupo de Investigación en Ingeniería y Nuevas Tecnologías Aplicadas (GRINTEC), Universidad Interamericana de Panamá, Panama City, 07095 Panama

**Keywords:** STEM, Educational robotics, BEAM, WEEE

## Abstract

In this work, BEAM robotics is proposed to enhance the STEM knowledge and skills of engineering students in the electrical, electronic, and mechanical domains. To evaluate the proposal, a course is designed and implemented based on a curriculum with objectives and learning activities centered on the design, construction, and operation of the BEAM robots. In addition, the connection between this proposal and computational thinking is explored. Students learn to recognize each part of robots and how they are related, abstract useful information from an electronic scheme and concretize it in a machine by systematizing their behavior. In addition, thanks to an evaluation of the behavior of the robot, identify the faults and apply the solution, as in the debugging process carried out in software programming. It should be added that BEAM robotics has a sustainable and low-cost aspect, which is used in learning activities where Waste Electrical and Electronic Equipment (WEEE) is recycled, and students are taught to value and integrate these parts into the design of the robots. A pre and post survey and a respective statistical analysis and evaluation of curricular activities are presented as evidence of the improvement observed in students’ STEM knowledge and skills. In general, the results show that this new teaching tool can promote the STEM curriculum in engineering students and motivate implementation, as a new educational robot, at other academic levels such as secondary and pre-secondary education.

## Introduction

A review of the state of the art indicates that educational robots motivate students to engage in STEM and serve as a very effective and efficient platform to improve the curriculum in these areas (Ucgul & Cagiltay, 2014). According to Papert (Wooster & Papert, 1982), educational robots are one of the best tools to implement the principles of constructivist teaching. Educational robots have become a trendy tool for teaching in all levels of education. Microprocessor-based educational robots and quick assembly kits are commonly used, which offer some morphological flexibility (various ways of building and operating it). Through their design, construction, programming, and execution, these robots have as fundamental objectives: to enhance STEM knowledge, develop critical thinking, engineering design, problem-solving, creative thinking, teamwork, engagement in science and technology, and reduce cultural and psychological barriers on all of these above topics (Barak & Assal, 2018).

In this didactic tool, the primary way of expressing their behavior to interact with the environment is by coding the user’s ideas through a programming language. This interaction is based on the user sending commands to the machine and associated with an optimal response as a measure of success that potentiates a curriculum firmly focused on programming. However, the highly concentrated focus on programming begins to put aside more intimate issues that robots offer to explore and are being ignored. For example, morphology (electromechanical system), physical operation of sensors and motors, electronic circuits, and energy optimization of the robot (Rihtaršič et al., 2016). These issues should not be relegated or trivialized. Instead, discovering what is in that “black box” should be encouraged; that is, the robot’s internal mechanical, electrical, and electronic parts and how they are related to each other. This knowledge can closely support the understanding of current technology based on electrical, electronic, and mechanical mechanisms. Likewise, with the rise of artificial intelligence and its deeper inclusion in teaching, robotics should not be limited to promoting primarily language-specific programming skills (Cox, 2021). Instead, the understanding of how software may be implicit in the concrete morphology of some electromechanical device; conversely, how to abstract the functioning of things from concrete reality and encode them cognitively. In other words, the morphological construction of a robot is a process that follows a particular algorithm from a previous design, that solves problems of interaction with the environment, such as perception of physical magnitudes to act accordingly, allowing mobility for self-preservation, and not only to fulfill a task specified by a user (Hasslacher & Tilden, 1995).

As an alternative to these challenges, in this work, BEAM robotics is proposed. BEAM is the acronym for the words: Biology, Electronics, Aesthetics, and Mechanics and was introduced in the nineties by Mark Tilden as unconventional and bio-inspired robotics (Hasslacher & Tilden, 1995). At BEAM, robots are not designed to accomplish specific tasks programmed to serve a user; instead, it is sought that the machines are autonomous and can interact in an unknown and hostile environment. Their goal is to live and preserve themselves and not depend on a human once built. The design, construction, and behavior of BEAM robots follow several criteria, such as a simple morphology without a microcontroller or microprocessor and made from simple analog electronic elements such as resistors, capacitors, transistors, diodes, light-emitting diodes (LEDs), cables, motors, and mechanical parts such as gears. The control of its behavior is achieved by a spiking neural network called nervous network (NV), built with simple analog electronic elements or basic integrated circuits(Hasslacher & Tilden, 1995). Integrated circuits can be used but keeping the most remarkable simplicity. Additionally, these items are encouraged to be obtained from discarded equipment, or so-called Waste Electrical and Electronic Equipments or WEEE (Cucchiella et al., 2015).

This approach has been little explored for educational purposes. In the literature review performed at the beginning of this project, only the following work of Ruiz del Solar (Ruiz del solar & Avilés, 2004) was found. In this work, the use of BEAM robots is presented in various workshops of a hundred children ranging from K7 to K10 to explore topics such as solar energy, the operation of motors, and other electrical, electronic, and mechanical elements. Although the results were quite encouraging, the idea of a curriculum with BEAM robots was not explored.

Commonly, educational robots, as a didactic resource for teaching–learning, can be used for three primary purposes (Muñoz Repiso & Caballero González, 2019):

1. The educational robot is the main object of learning, that is, as a resource for learning robotics.
2. The robot is a teaching–learning medium or tool—for example: to teach STEM or another area of knowledge.
3. The robot is a means to develop learning, skills, and abilities. Example: develop critical thinking, engineering design, problem-solving, creative thinking, teamwork.

The proposal presented in this work focuses on the last two objectives. It seeks to enhance the STEM curriculum in engineering students by developing skills and knowledge in various domains, such as electrical, electronic, and mechanical engineering, using BEAM robotics. The purpose is not to build the robot to perform specific tasks or enhance the programming of computer languages; instead, it proposes using the design, construction, and execution process as a didactic resource. That is, as it was expressed by Ruiz del Solar (Ruiz del solar & Avilés, 2004): “*Robotics is a way, not the end*.” In this way, BEAM robotics is used with an educational purpose, which through its design, construction and operation develops and improves knowledge and skills in electrical, electronics, and mechanics. Also, as a complementary topic, this tool is applied to improve computational thinking by supporting the concretization of algorithms, not in software but hardware. It is essential to mention that this educational robot has a sustainable and low-cost aspect since its parts can come from the recycling of discarded equipment.

Based on this new educational robot, a curriculum is designed and implemented in a course developed for university students from the Faculty of Engineering of the Universidad Interamericana de Panama. Pre and post-course surveys, learning and evaluation activities, and logbooks were developed to collect and analyze data. It is essential to mention that the course was virtual, designed and assembled in a Learning Management System (LMS), specifically, in the learning platform of the University: Moodle. However, kits with parts and tools were created for the construction of the robots. Thus the students were able to work from their homes, which facilitated the process during the COVID 19 pandemic.

A description of the initial status and background of the proposal was published in (Boya Lara & Vega, 2020). Rather, current work focuses on demonstrating how BEAM robotics enhance STEM knowledge and skills. In addition, it delves into the design of the course structure, the curriculum, and the research. The contributions of this paper are the following:

1. Development of a curriculum based on BEAM robotics for the teaching–learning of the following domains: Electrical, Electronics, Mechanics, Computational Thinking, Recycling of WEEEs.
2. Design and implementation of a new didactic tool for STEM teaching: The BEAM robot, a sustainable and environmentally friendly technological approach.
3. A study of the impact of the new educational robot on the STEM learning of university students.

## Related work

### BEAM robotics

Mark Tilden introduced BEAM robotics in the 1990s as a new form of non-traditional robotics, in which the robot is not designed to perform specific goals for human support. Instead, robots are built with the objective that they exhibit autonomous behavior and self-preservation in an unknown and hostile environment (Tilden, 1997). Tilden called these machines: biomorphic, a parallel form of life that does not have an “intelligence” conventionally. Since they do not have a complex processor and therefore cannot process an internal symbology to establish communication with humans, it is an analog computer that allows them to interact with the environment, move and survive. More than an entity with a functional purpose, it is a design based on biological paradigms (Rietman et al., 2003). Its computation is carried out in an analogous, modular way and is implicit in the morphology or skeleton of the robot. In this sense, a neural model, called NV (Nervous Network), works to express, and communicate signals in a bidirectional way while detecting external stimuli through sensors to move motors accordingly. The design and construction criteria for BEAM robots can be listed as:

1. The simpler, the better. A simple behavior and morphology.
2. The control is not based on a microcontroller or microprocessor.
3. Simple analog electronic elements are used for its construction, such as resistors, capacitors, transistors, diodes, light-emitting diodes (LEDs), cables, and motors. Also, mechanical elements such as gears. Integrated circuits can be used but keeping the most remarkable simplicity.
4. Its behavior is implicit in morphology and controlled by an analog and a modular NV. NV is based on a combination of transistors, diodes, resistors, light sensors, or simple integrated circuits.
5. To use as far as possible electrical, electronic, and mechanical elements extracted from discarded equipment or WEEEs. Recycling is encouraged by seeking sustainable, environmentally friendly, and low-cost designs.
6. The BEAM structure must imitate insects or some other biological entity and ensure good aesthetics and functional designs.
7. Robots must be powered as efficiently as possible. Typically, batteries with small voltages (3 or 5 V) are used. However, the use of solar energy converted by photovoltaic cells is encouraged.

**Fig. 1:**
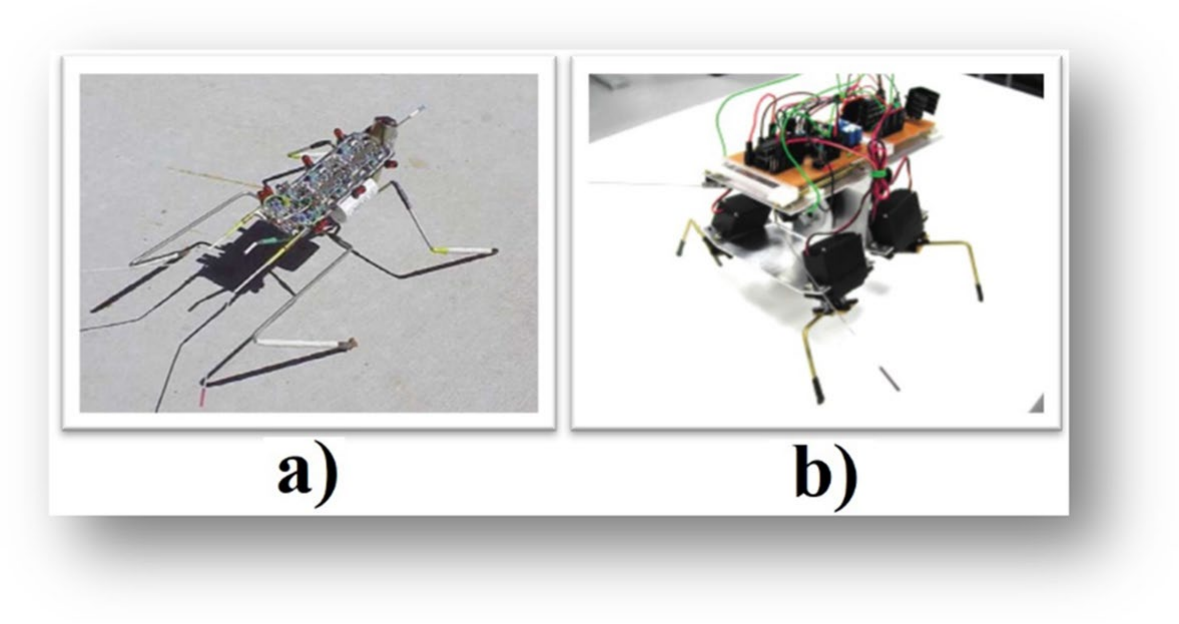
**a** BEAM robot with an insect-like shape (Rietman et al., 2003), **b** A BEAM robot, called QUL 1.4 (Vadakkepat et al., 2012)

Figure 1a shows an example of a BEAM robot in the shape of an insect called “Walker”, that has two motors and four legs that allows it to move, and, in addition, a front sensor to avoid obstacles. This robot starts its movement with little energy in its circuits and displays a sequential move powered by the NV connected to the motors. Figure 1b shows a BEAM robot called QUL 1.4 (an acronym for Quadrupedal Uncontrolled Locomotion), which has four NV neurons in a ring and four motors proposed in (Vadakkepat et al., 2012) as an upgrade for the Walker.

**Fig. 2:**
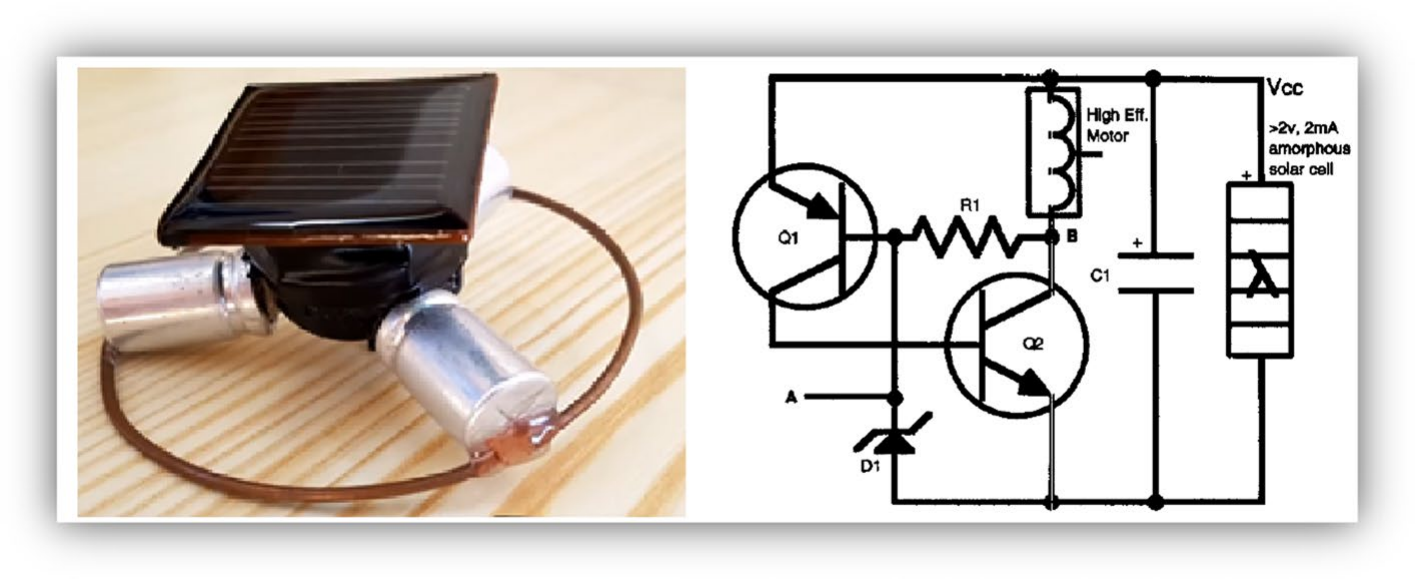
The BEAM SYMMET robot and its electronic circuit (Hasslacher & Tilden, 1995)

Batteries power these two previous robots, and although their design maintain the BEAM criteria, they require great experience, knowledge, and skills to build. There are simpler BEAM robots, such as the SYMMET and the PHOTOPOPPER. In Fig. 2, the SYMMET is shown. The image to the right of the robot is the electronic circuit. This robot uses solar energy, converted into electrical power by a photovoltaic cell, to charge capacitors (small tanks that store electrical energy). A combination of an electrical resistor, two transistors, and a LED forms the NV, which works by activating a small direct current motor when the set voltage threshold is reached. The motor, quickly receives the energy from the capacitors, making the robot move in small jumps; the hopping cycle increases or decreases depending on the amount of solar energy received and converted by the solar cell. If there is a good level of sunlight, the robot will move continuously, but if there is little energy, its movement will slow down until it stops if it is dark. It is possible to make the robot go from jumps to a continuous motion by controlling the resistance value. Once built, the robot is like a living being whose movement depends randomly on where it lands in the next jump.

**Fig. 3:**
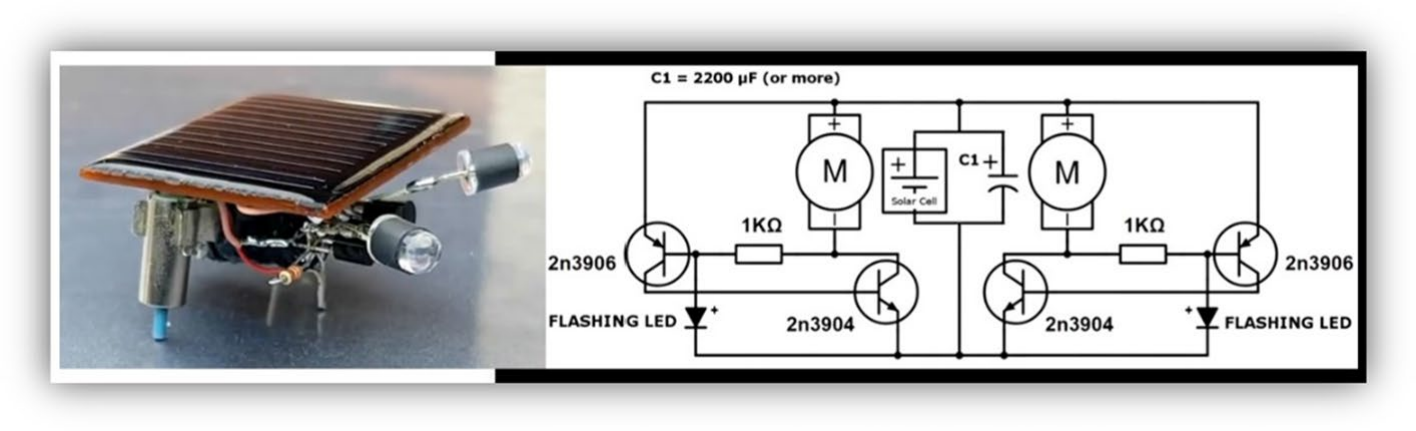
Robot BEAM Photopoper(Tasi Geri, 2017)

The Photopopper is another BEAM robot that presents simplicity in its construction and understanding. In Fig. 3, this robot is shown, which is the approximate combination of two SYMMET circuits. This design adds the ability to move in the direction where the light is most intense. In this case, the two NVs compete to activate its motor first, which depends on the intensity of the light reaching the left or right side of the robot. LEDs, like eyes, detect light and provide the voltage threshold to discharge and activate the motor, once an NV starts its motor, the robot moves in that direction.

### BEAM robotics and its relationship with STEM

Specific STEM-related knowledge and skills are applied to the construction of BEAM robots. To meet these goals, the design and construction of robots must integrate the knowledge of biology, mechanics, electronics, control systems, artificial intelligence, mathematics, and sequential or algorithmic processes (Rietman et al., 2003). The proper functioning of these robots requires that students follow the criteria on which BEAM robots are based, both in construction and operation. In this way, the following technical knowledge and skills are needed: construction using electronic schematics, electronic welding, electrical risk assessment, energy sources, handling of electronic and mechanical instrumentation. In addition, fault detection, search, and investigation of technical details of electrical and electronic elements. BEAM robots, promote these skills and have the implicit potential to support the STEM curriculum. Previous studies on educational robotics with similar characteristics have shown an improvement in STEM knowledge and skills (Laut et al., 2015; Rihtaršič et al., 2016; Ruiz del solar & Avilés, 2004; Sullivan & Heffernan, 2016; Tuluri, 2017).

SYMMET and PHOTOPOPER are used in the course due to their simplicity. In this case, the student aims to identify and relate, in an intuitive way, the function of each electromechanical element in the designs. Also, issues of electricity and electronics will be addressed in the initial stage of the construction and subsequent execution of the robots. It is expected that if the robots do not work, the builders will have to perform a process of inspection and troubleshooting or “debugging,” like those carried out in computer language programming but focused on hardware. Failure is an expected and welcomed event in the course, as trial and error foster skills for science and engineering (Shute et al., 2017).

### BEAM robotics and WEEE

**Fig. 4:**
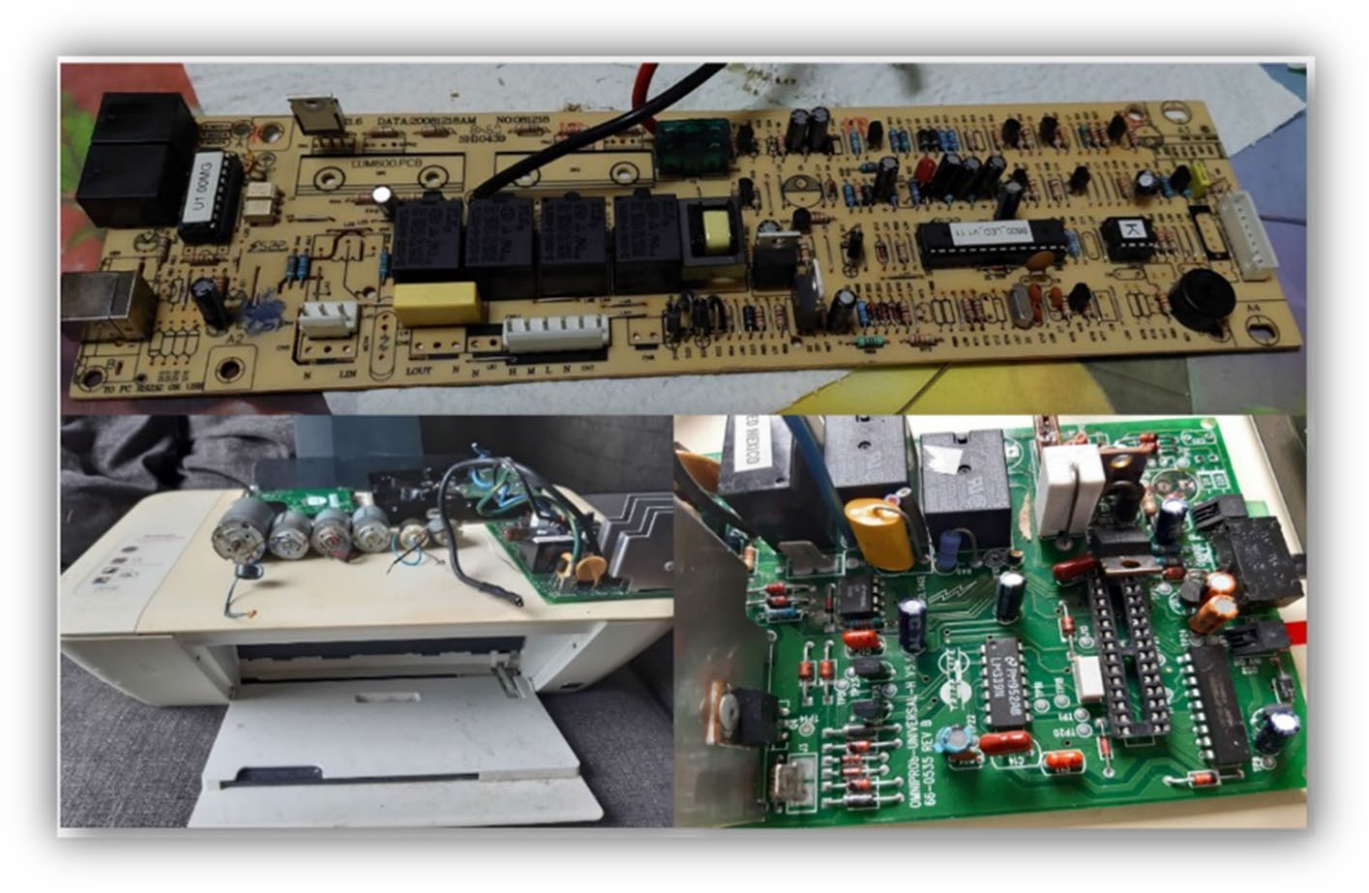
Above, the motherboard of an uninterruptible power supply (UPS). Bottom left, a printer with typical motors recovered from this equipment and next to it, the board of an audio amplifier—all equipment discarded due to malfunction or end of life

With BEAM robotics, the equipments known as WEEEs are valorized, a waste that grows steadily every year, being a major environmental problem (Ilankoon et al., 2018). Although it does not seek to solve this problem, BEAM robotics promotes the recycling of WEEE, attributing added value and a didactic property to it. WEEE in BEAM robotics results in low-cost designs, simplicity, and a sustainable teaching resource (Klapyta, 2014). This feature allows robots to be built with easily recyclable elements from WEEE (Tilden, 1997). Some examples of disposal equipment that generally accumulate in homes, schools, government entities, industries, and businesses, as shown in Fig. 4, include: uninterruptible power supplies (UPS), printers, and audio amplifiers. These WEEEs are taken to municipal landfills, where they accumulate without any purpose other than polluting the environment. From the printer shown in Fig. 4, motors in good condition can be extracted, as well as other elements such as resistors, capacitors, LEDs, transistors, etc. All these WEEEs contain many parts that can still be used to construct or repair other electronic devices.

The proposal integrates this into the BEAM robotics learning course and adds evaluation activities focused on recycling WEEEs and enhancing the design of robots with recovered elements. It is a didactic environment that offers students an enriching aspect of the technologies they use daily. A more intimate look at that “black box” allows understanding its parts and their relationship, even giving them a new use that can stimulate creative thinking.

### BEAM robotics and computational thinking

The relationship with programming languages and algorithms, is explored in the proposal. Computational thinking is usually related to coding or programming; however, it has more significant cognitive implications. When the analysis of a system is approached, it is important to abstract each of its elements, instead of viewing the system as a whole. In this way, it is more feasible to characterize its functionality, how it is internally related and the way it interacts with the environment. A learning activity seeking to enhance computational thinking, should encourage the abstraction of the elements of a system and the algorithmic understanding its functioning. Like programming, users input data, the system processes it, makes decisions according to specific criteria, and generates outputs as optimal responses (Shute et al., 2017). The proper construction and functioning of BEAM robots follow a procedure, like an algorithm. The BEAM robot acts through an analog computer that is directly connected to the morphology of the robot. Any anomaly in this conformation can change the robot’s behavior; for this reason, it is necessary to understand each element and how they relate to each other.

Another topic of computational thinking is problem solving, generalization, and trial and error (Bers et al., 2014). As we have explained previously, in the construction of the robots, it is expected that there will be failures in the building and the execution. These challenges motivate troubleshooting and debugging, as well as proposals to improve the design and build process. All the experience acquired in redesign, construction, and debugging promote the construction of more complex entities; and, thus, the generalization of thought and knowledge.

## The BEAM robotics course

The proposal’s objective is to evaluate whether a curriculum based on the design, construction, and operation criteria of BEAM robotics, develops and improves STEM knowledge and skills in engineering students. In this way, a course was designed and implemented for engineering students from the Universidad Interamericana de Panamá to evaluate this objective. It is essential to mention that the financial resources for the course and research were obtained through a Public Call for Educational Projects in Science and Technology 2019 National Secretariat of Science, Technology, and Innovation of Panama (SENACYT). A proposal was made to the call “Development of an experimental course in basic robotics using electrical and electronic waste,” which obtained the funds. Initially, it was face-to-face; however, due to the COVID-19 emergency, it was adapted to a virtual format. The modules were designed and assembled in a Learning management system (LMS), specifically, in the learning platform of the University: Moodle. It is essential to mention that the course is not part of the engineering degree curriculum; it is an extracurricular course.

**Fig. 5:**
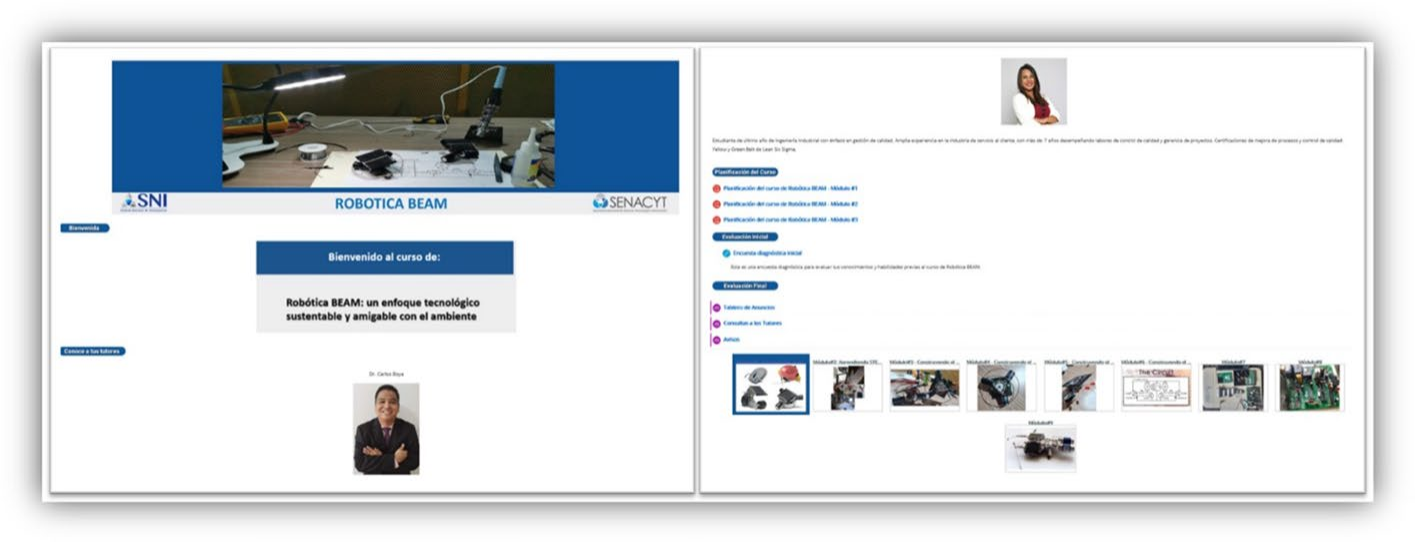
Images of the BEAM robotics course in Moodle. The welcome is shown with the tutors and the nine modules, each with a specific learning topic

Figure 5 shows an image of the presentation of the course. It is divided into the title, a brief introduction of the tutors, the course planning, the pre and post-survey, a bulletin board, and a blog for consultations with the tutors. At the end, access to the nine modules of the course. The course was called: “B*EAM Robotics: a sustainable and environmentally friendly technological approach.”*

Once the administrative and financial procedures were carried out prior to the development of the course, a study of the state of art was conducted to determine the guidelines of the course. The works (Becker et al., 2014; Cucchiella et al., 2015; Hasslacher & Tilden, 1995; Rihtaršič et al., 2016; Ruiz del solar & Avilés, 2004; Tuluri, 2017; Zhao et al., 2016) were identified and used as a reference. Table 1 shows the contributions of these works to the proposal, such as design criteria and construction of BEAM robots, use of these robots as a tool for teaching–learning, STEM and educational robotics, design of the STEM curriculum based on educational robotics, and research design for the integration of new educational tools for STEM in engineering students—also, concepts about WEEE and recycling.

**Table 1:** List of works used as a reference for the design of the course, the curriculum, and the investigation

References	Paper name	Topic contributed to the proposal
Hasslacher and Tilden (1995)	Living machines	BEAM robotics fundamentals and criteria
Ruiz del solar and Avilés (2004)	Robotics courses for children as a motivation tool: The Chilean experience	BEAM robots as a teaching–learning tool
Tuluri (2017)	Robotics in STEM Education: Redesigning the Learning Experience	Integration of robotics into the STEM curriculum and design of STEM experiences with robotics
Rihtaršič et al., (2016)	Experiential learning of electronics subject matter in middle school robotics courses	Educational robotics for the development of skills and abilities in electronics, curricular design, and research
Becker et al., (2014)	Project circuits in an introductory electric circuits course	Use of project-based learning for teaching electrical circuits in engineering students
Zhao et al., (2016)	Android-based mobile educational platform for speech signal processing	Development and research on a new platform for teaching electrical engineering students
Cucchiella et al., (2015)	Recycling of WEEEs: An economic assessment of present and future e-waste streams	State of the art of different WEEE streams and sustainable management

**Fig. 6:**
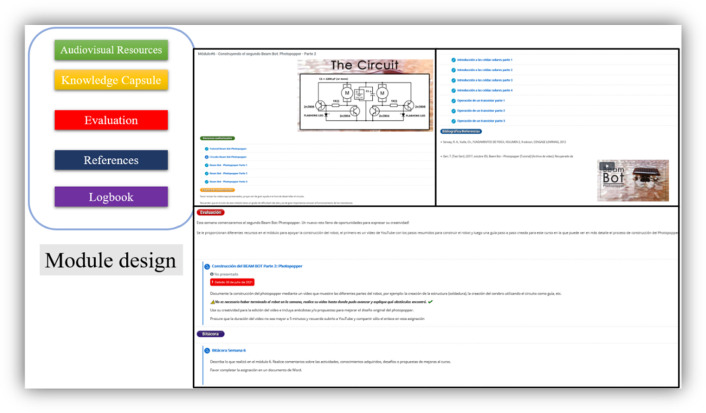
Module #6 is shown: “Building the second Beam Bot: Photopopper—Part 2”. As can be seen, it is organized by five tags: Audiovisual Resources, Knowledge Capsule, Evaluation, References, and Log. Students have access to video tutorials, evaluation activities, and the possibility of relating their experiences in a blog

**Fig. 7:**
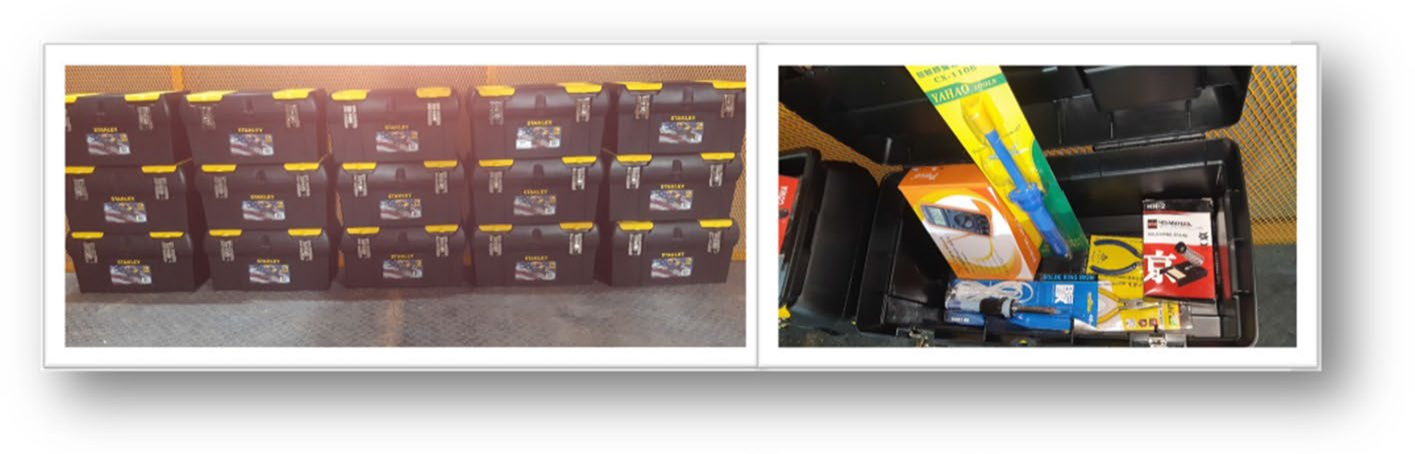
The figure shows the kit with its box, which contains: two resistors, four capacitors, three motors, five LEDs, two solar cells, a multimeter, soldering iron, desoldering pump, soldering cable, tip and cutting pliers, a rubber gun, and cables. The kits were given to the students to make the robots in their homes

The course followed these guidelines:

1. A duration of nine weeks; one module per week, for a total of nine modules. At the end of each week, on Saturday, a synchronous session with the students to clarify doubts, make comments, and receive more information about the course from the tutors. This session usually lasts one hour.
2. Two BEAM robots are proposed: The SYMMET and the PHOPOPPER, mainly due to their simplicity in construction and execution. In addition, the robots are powered by solar cells, and the materials for their construction can be recycled.
3. Progressive learning, in which the first two modules cover essential topics of electricity and electronics, and it is in the third module where the construction of the first BEAM robot begins: the SYMMET, the simplest.
4. The course is automatic; a level of autonomy is expected from the students, so there is only one synchronous session per week of at least one hour. Each module follows the design shown in Fig. 6, with five labels to organize it: Audiovisual Resources, Knowledge Capsule, Evaluation, References, and Logbook.
  - **Audiovisual Resources**: Video guides for the various learning activities. Most of these videos were created by the tutors.
  - **Knowledge Capsule**: Brief papers or videos that explain the concepts or meanings of each topic of the module.
  - **Evaluation**: Assessment activities that the student must deliver, such as an exam, essay, or a video.
  - **Reference**: References to the written or multimedia material that has been used to support learning.
  - **Logbook**: In this section, a document must be uploaded with the lessons learned, experiences, problems, comments, criticisms, or other topics that the student believes pertinent.
5. A kit is developed with the number of parts and tools necessary to construct the two robots. Figure 7 shows this kit with its box, which contains: two resistors, four capacitors, three motors, five LEDs, two solar cells, a multimeter, soldering iron, desoldering pump, soldering cable, needle-nose and cutting pliers, a rubber gun, and cables. The kits were given to the students to make the robots in their homes.
6. Two modules, #7 and #8, are dedicated to the recycling of WEEE. Figure 8, a video tutorial showing how to extract elements, such as resistors, capacitors, LEDs, transistors, motors, and gears. Afterward, the students are invited to integrate these parts into the BEAM robots and, if possible, redesign the robots, keeping the BEAM criteria.

**Fig. 8:**
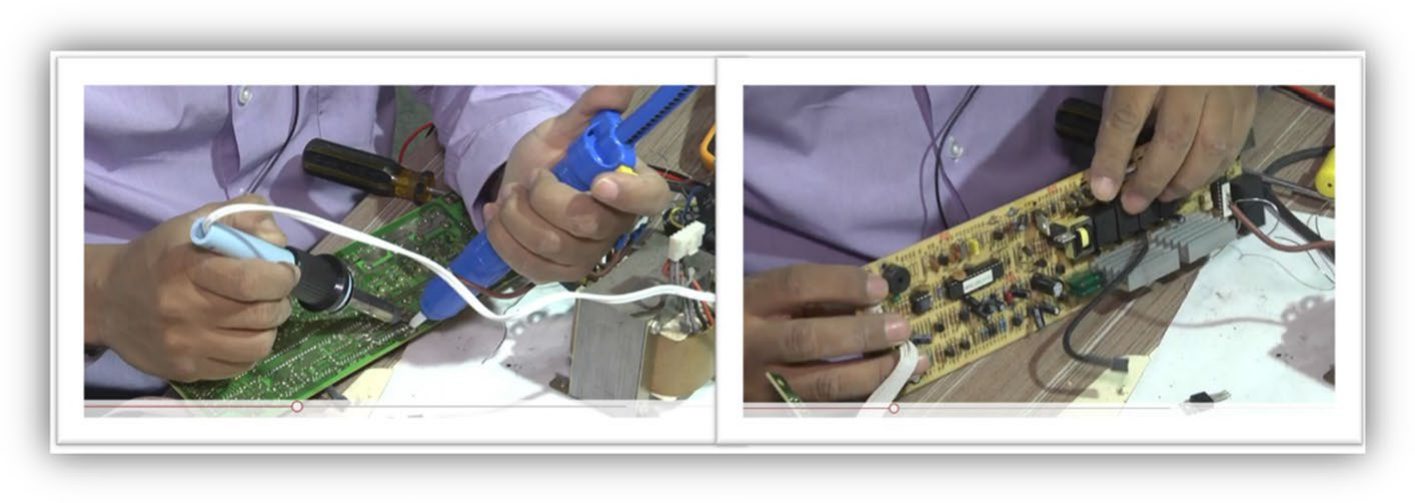
Images of a video tutorial developed for the course where it is taught how to recycle through the extraction of electronic parts, in such a way that they serve for the construction of BEAM robots. In addition, the video encourages students to value WEEE

### Curriculum design and its implementation

**Fig. 9:**
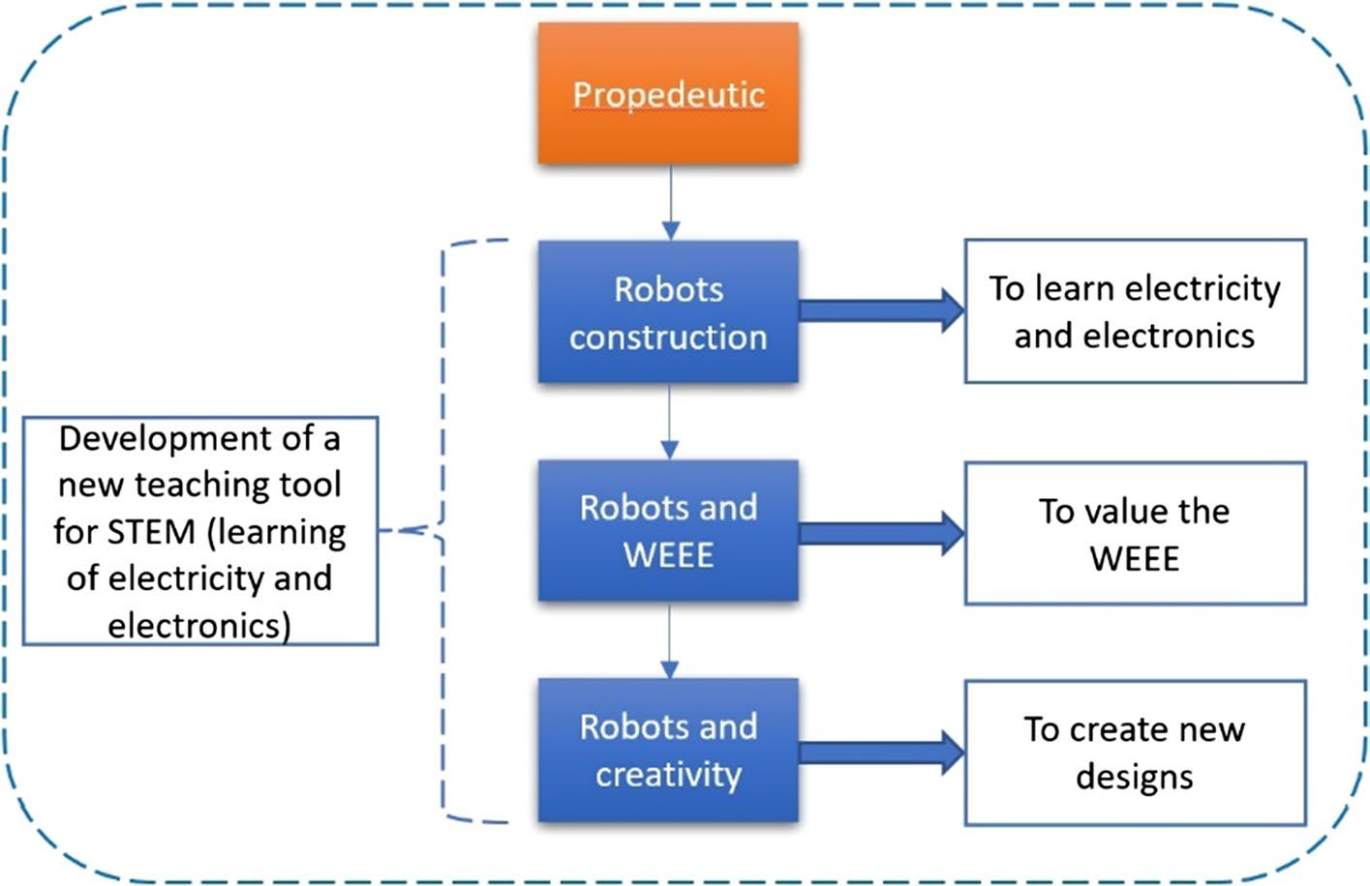
Proposed stages of the BEAM course curriculum for the development and improvement of STEM skills and abilities

The course curriculum design is oriented in three phases, as can be seen in Fig. 9: Robots Construction, Robots, and WEEEs and Robots and creativity. In addition, a propaedeutic phase in which the student performs learning activities focused on recognizing, understanding, and interpreting concepts, definitions, terms, relationships, and procedures in electricity, electronics, and mechanics. The Robots Construction phase is focused on the application of the learning of the preparatory phase and the development of concrete skills such as electronic welding, interpretation of electronic diagrams, and concretization through the construction of the robot. The next phase, Robots, and WEEEs seeks to teach how to recycle WEEE. It is taught how to extract parts and evaluate them for possible integration into the robot. Also, in the development of computational thinking. The final phase is intended to stimulate creativity through a robot design activity.

The curriculum was designed with a constructionist approach, “learning by doing” (Papert & Harel, 1991). In this way, the following are used as learning strategies: active learning, hands-on experiences, problem-solving, and project-based learning. Students are expected to build knowledge and develop the right skills based on these strategies (Altin et al., 2013; Danahy et al., 2014). As previously indicated in the course design, it was decided to implement nine modules (1 for a week) dealing with different topics, learning objectives, and evaluation activities, as shown in Table 1. Depending on the learning objective, each module was designed to deal with different dimensions of knowledge. In this case, by the three types from the review of Bloom’s Taxonomy (Anderson et al., 2001): Factual, Conceptual, and Procedural.

Factual knowledge deals with the concepts and meanings that students must acquire, identify, or recognize in a specific area. Example: Modules #1 and #2 have a propaedeutic approach mainly. The learning objectives focus on students identifying and recognizing the fundamental topics in electrical and electronic engineering, such as electrical resistance, current, voltage, energy sources, and electrical hazards. Conceptual knowledge is about the interrelationships between the essential elements forming a larger structure to function together. In module #1, students build electrical circuits using a simulator and are asked to change resistance or voltage values and measure current. There is a relationship between these three variables, called Ohm’s Law, and students are expected to infer this relationship with the learning activity. The previous knowledge, procedural, is related to how the student uses the prior knowledge. The ability to identify what relationship, understanding, or skill is appropriate for a specific task. Module #5 is a perfect setting for the development of this knowledge. It is focused on the construction of the Photopopper, a robot that requires the ability to weld, identify elements and operation, and perform electrical measurements. In addition, abstract the connections of the components from a schematic following a procedure. It is important to emphasize that the development of this knowledge is not specific to each module; rather, it depends on the learning objective and the cognitive process to be developed.

As explained, modules #1 and #2 have an introductory and propedeutic approach. Modules #3 and # 4 deal with the construction of the first robot: the SYMMET. The learning strategy, in this case, was to use the robot construction process to teach various electronic elements: the capacitor, the diode, the LED, and the transistor. And also to train in electronic welding. The robot's operation depends on connecting all these parts correctly and following the electronic circuit of the robot. The students were asked to try the process as many times as necessary until the robot worked adequately. To assess learning, they were asked to make a video about this construction process. In the sessions, feedback was carried out. It is important to note that the objective of this activity was not for the robot to work perfectly and look the same as the videos; instead, it uses the construction process to develop STEM skills and knowledge. The following two modules, #5 and #6, respectively, were focused on the construction of the PHOTOPOPPER. This robot is made up of two circuits, the same circuit used in SYMMET robot, but connected in a way that makes them compete with each other to energize the motor on their side first and make the robot move in their direction (see Figs. 4 and 5). In this manner, it was expected to reinforce the skills and knowledge acquired in the construction of the SYMMET. The PHOTOPOPPER has greater mechanical and aesthetic complexity, which represents a more significant challenge for students. As an evaluation activity, the students were asked to explain the construction process and develop a robot's operating procedure flowchart. The objective was to evaluate if they could create an algorithm that could be programmed into a computer and associate this activity to computational thinking.

Modules #7 and #8 had two objectives: to learn to recycle from waste equipment and assess WEEE. Video Guides were made disassembling an Uninterruptable Power Supply (UPS) and a printer. In the first of these modules, it was shown how to extract elements such as resistors, capacitors, diodes, LEDs, transistors, and others, using electronic welding equipment. While in module #8, the printer was used to demonstrate how to remove motors and light sensors. As an evaluation activity, the students were asked to apply this knowledge by recycling, using the UPS given to them by the University for this module or using their own WEEE. Later on, the module, the students were tasked to assess whether it was possible to use the pieces they had extracted in the construction of the robots.

The last module is focused on creativity as the maximum cognitive process (Anderson et al., 2001). It was expected that after the learning process of the eight modules above, the students would have the skill and knowledge to reformulate one of the two robots; also integrate parts extracted from some WEEE. In the last session, in addition to the video of the construction process, the students were asked to present their work to the tutors, other colleagues, and some other invitees during the end of course meeting. It is essential to highlight that each module contained an evaluation activity: a Logbook, in which the students were asked to relate their experiences, problems, criticisms, and any other topic that they wished to express. As will be explained in the research design section, this was a fundamental tool for obtaining information about the student's learning process.

### Research design

In the proposal of this work, the course and its curriculum are designed to determine if BEAM robotics develops and improves knowledge and skills in five dimensions of STEM. A data collection and analysis strategy is developed to evaluate this objective, consisting of a pre and post course survey, analysis of the evaluation activities, and an interview at the end of the course.

For the selection of students, a call was made, which only 56 students answered from a universe of more than 800 engineering students. In addition, it was desired to have an inclusive course, so an attempt to motivate female students was made; however, out of the 56 applicants, only ten were women. Students from various areas of engineering responded to the call, all from the area of new information technologies: Engineering in data networks, Engineering in computer systems, Engineering in Electronics and Communications, Industrial Engineering, and Computer Technicians. It is important to mention that there are no mechanics, robotics, energy, electrical, or civil construction degrees in the engineering faculty. After interviewing the candidates and conducting another survey, 15 students were selected (60% male, 40% female), ages from 19 to 43 (Mean = 28.8, SD = 7.79). The objective of the second survey and the interview was to try to identify and select students who would commit to staying until the end of the course. The dismissal was a matter of great concern. Some students thought that BEAM Robotics was another traditional microcontroller-based robot; when it became clear that this robotics were not based on these devices, they decided to not participate in the course.

To evaluate students’ knowledge and skills before and after the course, a pre-and post-survey was applied with a five-point Likert-type scale (Tseng et al., 2013), ranging from 1 (strongly disagree) to 3 (neutral) to 5 (strongly agree). Table 2 shows the questions divided into five knowledge domains: Electrical, Electronics, Mechanics, Computational Thinking, and Recycling of WEEEs. The other tool for obtaining data were the evaluation activities, in which the videos were analyzed, and the learning objectives were verified. In addition to these, the Logbooks, in which students at the end of each module, wrote about the lessons learned, experiences, problems, comments, and criticisms, were also analyzed.

**Table 2:** STEM Knowledge and Skills Pre/Post Survey

Domain	Survey questions
Electrical	I can explain Ohm’s law
	I can indicate the value of an electrical resistance just by looking at it
	I know the difference between alternating and direct current
	I can measure voltage and electrical current
	I can measure how much energy an electrical capacitor store
	I can build an electrical or electronic circuit capable of exerting control
	A photovoltaic cell converts solar energy into electrical energy
	As the angle of incidence of light in a solar cell change, the amount of electrical energy converted changes
Electronic	I identify the symbols of a transistor, diode, capacitor, resistor, LED, photovoltaic cell, and motor
	Transistors and diodes are semiconductors
	I can calculate how much current a LED can receive without being damaged
	I feel able to weld with tin and iron
	I can unsolder an electronic piece of equipment
	I can identify the polarity of a LED just by looking at it
	I can solder using an electronic circuit as a guide
Mechanics	The direction of rotation of a direct current electric motor depending on the polarity of the power source
	An electric motor transforms electrical energy into mechanical energy
Computational thinking	I can explain what an algorithm is
	I can describe a process involving mechanical, electrical, and electronic elements using an algorithm
	I can program the behavior of a BEAM Robot in a programming language
Recycling of WEEEs	I can recover from electronic equipment, elements such as a resistor, a capacitor, a motor, etc
	Disposal equipment can be totally or partially reused

## Results

### Pre-and post-survey

This study assumes that BEAM robotics can positively impact the knowledge and skills of the following domains: Electrical, Electronics, Mechanics, Computational Thinking, and Recycling of WEEEs. A pre and post-survey using the Likert scale from 1 to 5 was used to determine this. As shown in Table 3, the students averaged around three on the pre-survey scale, indicating a basic knowledge in these domains. It is important to highlight that the student sample was taken from the Faculty of Engineering, which explains this level of knowledge. The Domains that most indicated confidence were the recycling of WEEEs (*Mean* = 3.567), Computational Thinking (*Mean* = 3.289), and Mechanics (*Mean* = 3.233). The Likert scale values of the post-survey yielded a value of 4324, which indicates an improvement in knowledge and skills in the five domains. The maximum value was for the recycling of WEEEs (Mean = 4.6) and, in general, all the other domains above a mean of 4.3, except Computational Thinking (Mean: 3.733), where this value did not differ much from the pre-test.

**Table 3:** Descriptive statistics (mean, standard deviation) of pre and post-survey about the knowledge and STEM skills in the domains: Electrical, Electronics, Mechanics, Computational Thinking, and recycling of WEEEs

Domain	Pre-test		Post-test	
	***Mean***	***SD***	***Mean***	***SD***
Electrical	3.025	1.531	4.325	0.862
Electronic	2.914	1.153	4.467	0.748
Mechanics	3.233	1.716	4.433	0.679
Computational thinking	3.289	1.424	3.733	1.338
Recycling of WEEEs	3.567	1.524	4.6	0.621
Five domains	3.093	1.532	4.324	0.906

Table 3 presents a Paired-Samples t-test analysis to assess the impact of the course on students and whether there is a change in STEM knowledge and skills. The results indicate that the students presented a significant difference, in general, evidenced in the value for t = − 12.556 (p < 0.05) for the five domains with a change in the mean of − 1.23. The domains that improved the most were Electronics and Electrical. However, Computational Thinking presented minimal improvement and with a p > 0.05 for the t-test analysis.

### Evaluation activities and Logbook

**Fig. 10:**
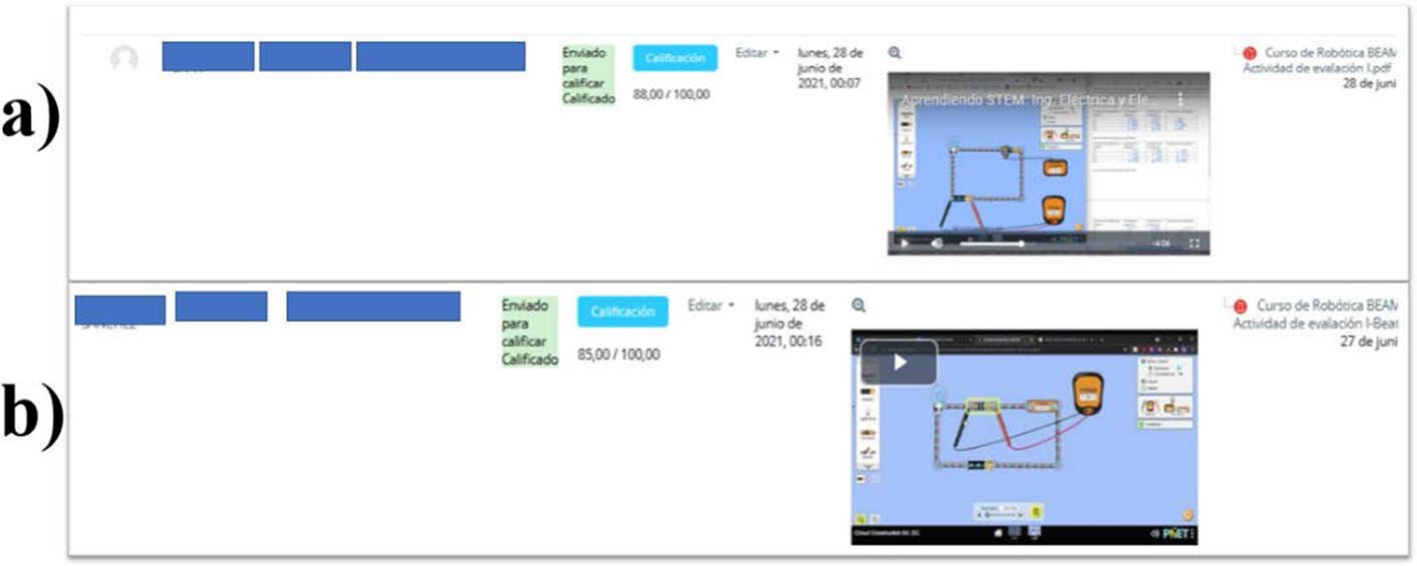
It is shown the evaluation activities of two students on the platform and their videos. **a** The activity “Construction of electrical circuits using the Phet Colorado Simulator Circuit Construction Kit: DC—Virtual Lab.” **b** The activity “Measuring current, voltage, and resistance, as well as understanding how they are related.” The personal data of the students have been covered with blocks in blue

All the students carried out the evaluation activities and developed their logbooks relating to their experiences and learnings. Recalling the curriculum activities. the first two modules had a preparatory objective: recognizing electronic, electrical, mechanical parts and related ones. The students recorded videos explaining the construction of a circuit, the effect of changes in values, and the relationship between different quantities, such as current, resistance, and voltage. Figure 10 shows one of the evaluation activities of two students on the platform. As mentioned in the curriculum, the Phet Colorado simulator (Rouinfar et al., 2002) was used. In the Logbook, everyone generally expressed their satisfaction with the knowledge gained in the two modules. One of the students suggested this learning approach should also be applied to different areas.

**Fig. 11:**
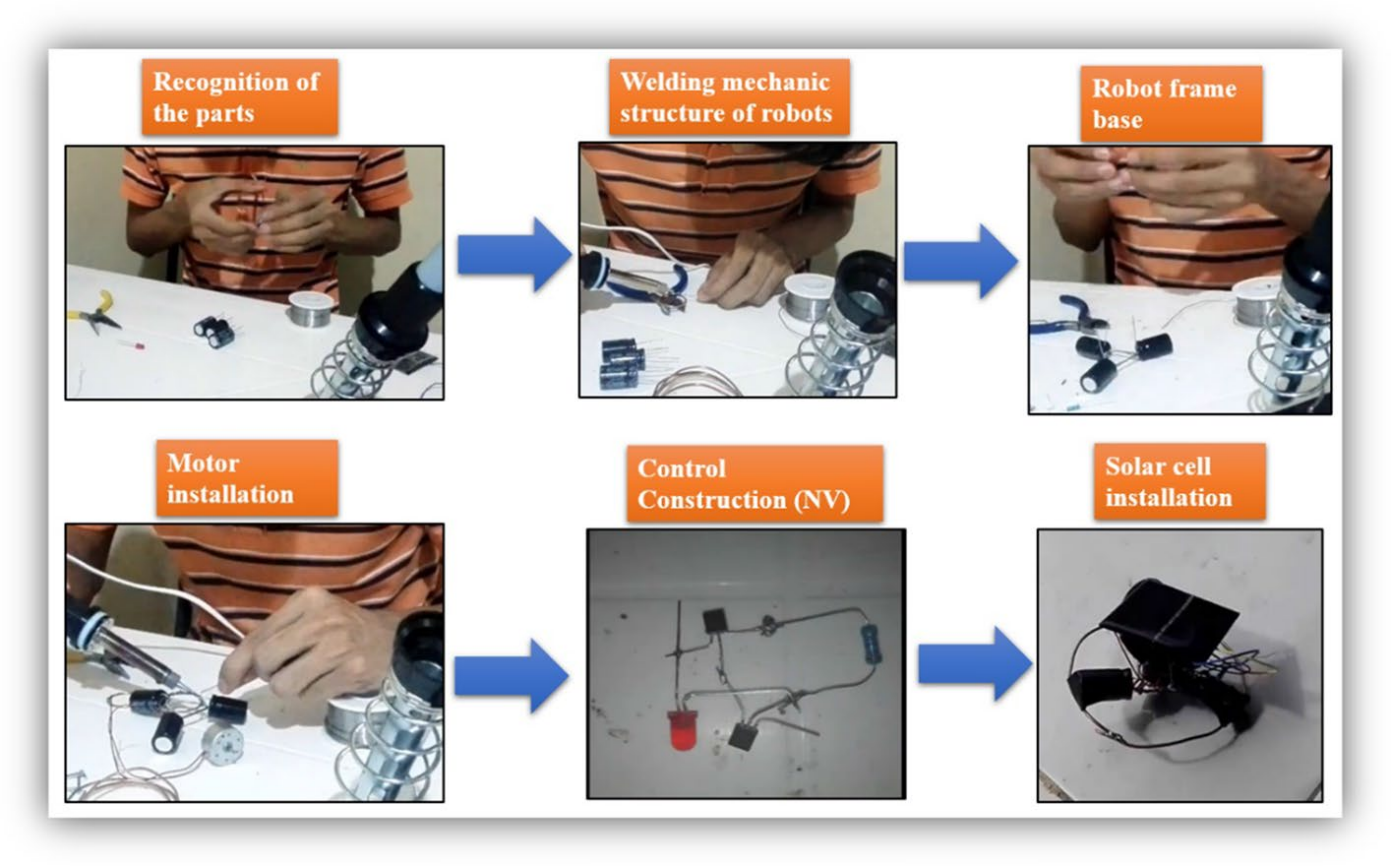
SYMMET robot construction process followed by all students

The following modules, #3 and #4, dealt with the construction of the SYMMET robot and the learning objectives that sought to apply the knowledge of the propaedeutic phase, but now concretizing it in the process of building the robot. In addition, the detection of failures due to errors in this process. In general, all the students made the robots following the video guides. In the initial sessions, they were encouraged to try another way to go about the construction process, and no variation to this construction pattern was observed. Figure 11 shows images of the video made by one of the students, which shows the process that all the students followed to construct their first SYMMET robot. From left to right and from top to bottom: recognition of the parts to be used; development of the robot structure with copper wire and capacitors and engine installation; construction of the robot control (neural network) with a LED, two transistors, a resistor, and aluminum cables. And finally, the integration of all the components and the solar cell. After this process, the students tested the robot with natural and artificial light. If the robot did not move, the student returned to the worktable. It is essential to indicate that only 3 of the students said they had experience welding (20%) in the pre-test survey, and none were female. After the course, all the students integrated this skill into their experience.

**Fig. 12:**
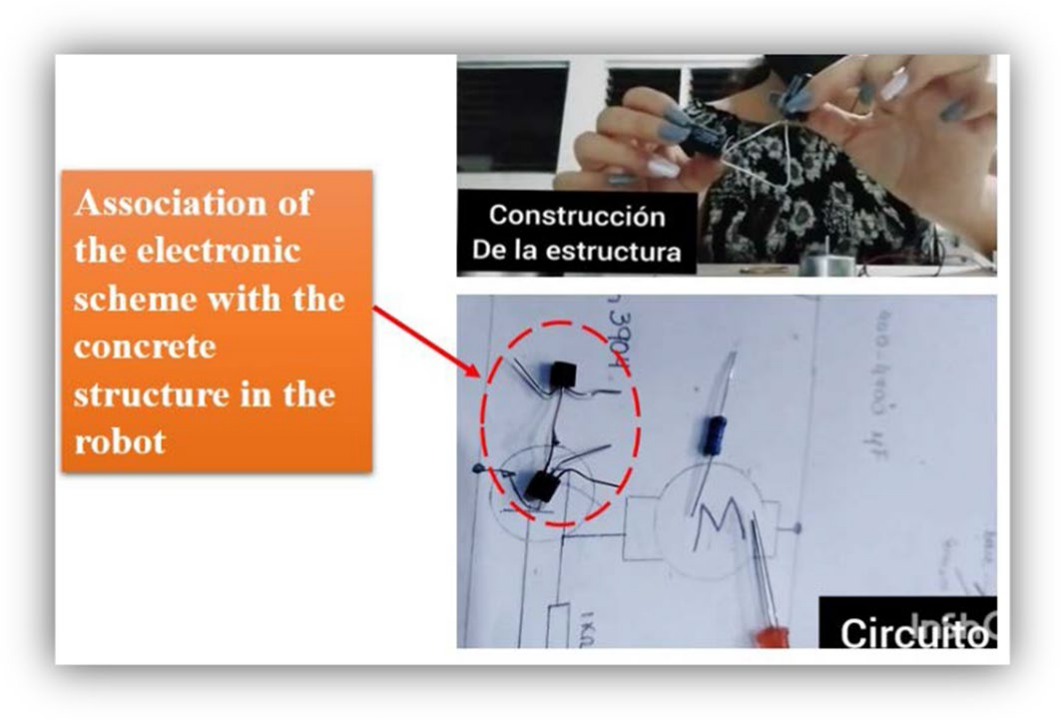
Images from the video of one of the six students that included the electronic schematic. Activity “Video of the construction of the SYMMET, commenting step by step.”

In addition to the process described, six students integrated the electronic scheme into the part recognition phase and explained how each part was related to the robot and its function. In this way, they recognized and identified the relationship between each part of the electrical circuit, and it was easier for them to solve fault problems. It is important to add that this was not in the guide of the videos, and it was something spontaneous in these students. Figure 12 shows images of the video of one of the students where the construction process with the scheme is explained.

In the Logbook, the majority (12 students or 80%) indicated no previous experience, neither welding nor building circuits guided by an electronic scheme. However, after the two modules, they felt confident working with new challenges involving the construction of real circuits and also observing how the SYMMET robot moved after a series of trials and errors. It was a very satisfactory experience and encouraged them to think about improving the robot. That is to say, that it moved faster or had a better aesthetic appearance. Four of the six women reported their lack of experience with electrical and electronic equipment and their great initial difficulty working the robot. However, they finished the activity at the end of the module and indicated that they had gained new knowledge.

**Fig. 13:**
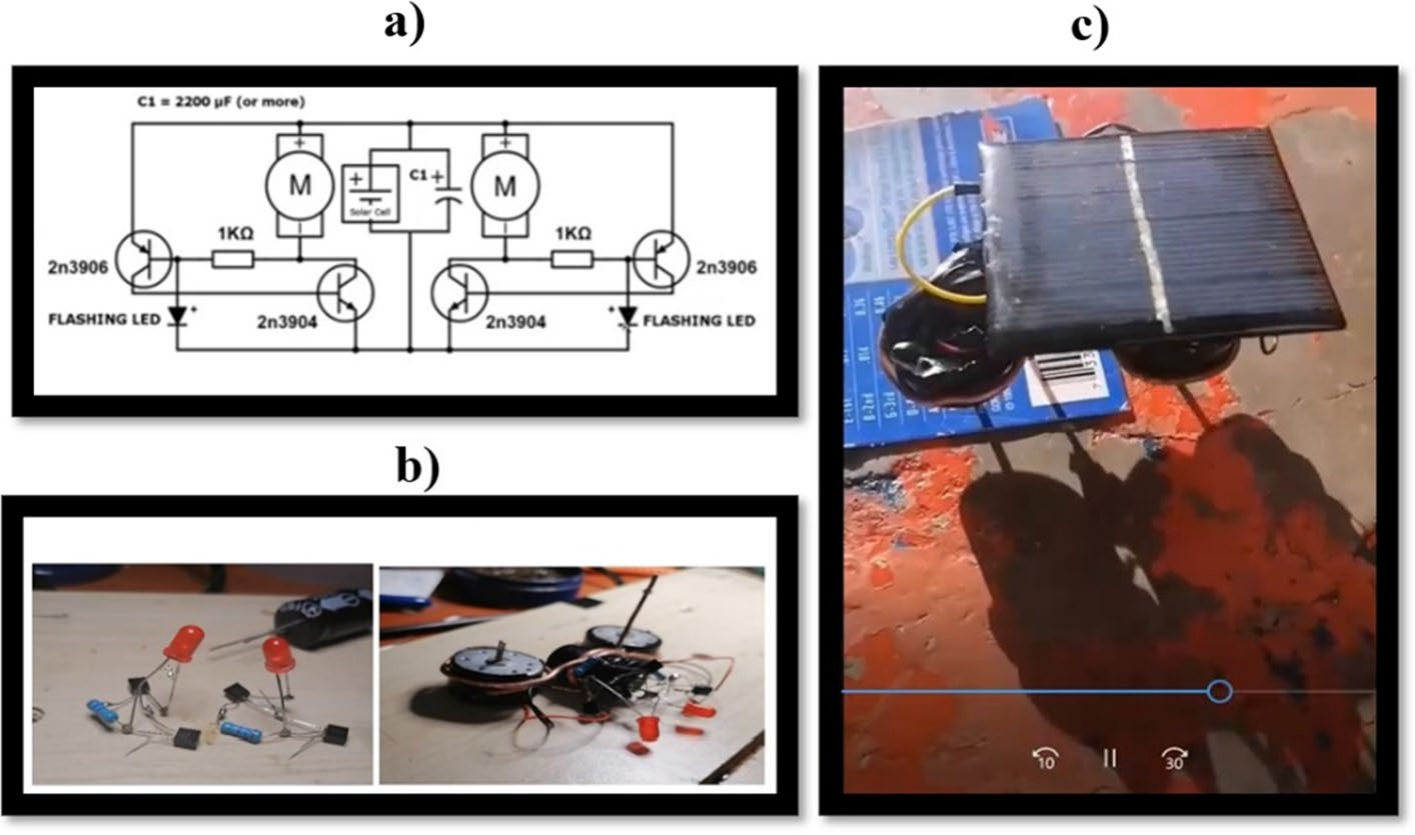
Images from the video of the “construction of the PHOTOPOPPER robot” activity. **a** Student explaining how the robot works and associating it with the electronic scheme. **b** Explanation of the two NVs and their relationship with the motors for movement to the left and the right, respectively. **c** Explanation of the robot's operation in an environment with artificial light

Modules #5 and #6 were dedicated to constructing a second robot: The PHOTOPOPPER. This robot presented a more complex challenge in construction since it simulates an insect, which can orient itself to where the light is most intense. In the evaluation activities, in addition to developing prior knowledge and skills, the objective was for the students to describe and explain the robot's operation as an algorithm. None of the students managed to make the flow diagram requested; however, in the videos, they were able to explain the process but used the video as support instead. In Tables 3 and 4, the domain Computational Thinking, was the one that presented the least improvement and remained approximately between 3289 and 3733 on a scale of 5. In the discussion section, we will address this issue more in-depth. However, all the students managed to build the robot and put it into operation. Figure 13 shows the images of one of the students explaining the construction and operation of the robot. As shown in the diagram, this robot comprises two NVs, which compete to acquire the energy stored in the capacitor from the solar cell. All students explained this operation and used the correct technical terms.

**Table 4:** Paired-sample t-test to evaluate the difference between the student pre-test and post-test

Domain	Paired differences (Shift mean)	*t*
Electrical	− 1.300	− 8.106
Electronic	− 1.552	− 9.423
Mechanics	− 1.200	− 3.562
Computational thinking	− 0.444	− 1.526
Recycling of WEEEs	− 1.033	− 3.439
Five domains	− 1.230	− 12.556

At the 5% significance level, the data provides sufficient evidence to conclude that the mean of the post-test is higher than the mean of the pre-test with exception computational thinking domain that presented  a p > 0.05 (p = 0.065), so that there was no significant change between the pre-and post-test for this domain

**Fig. 14:**
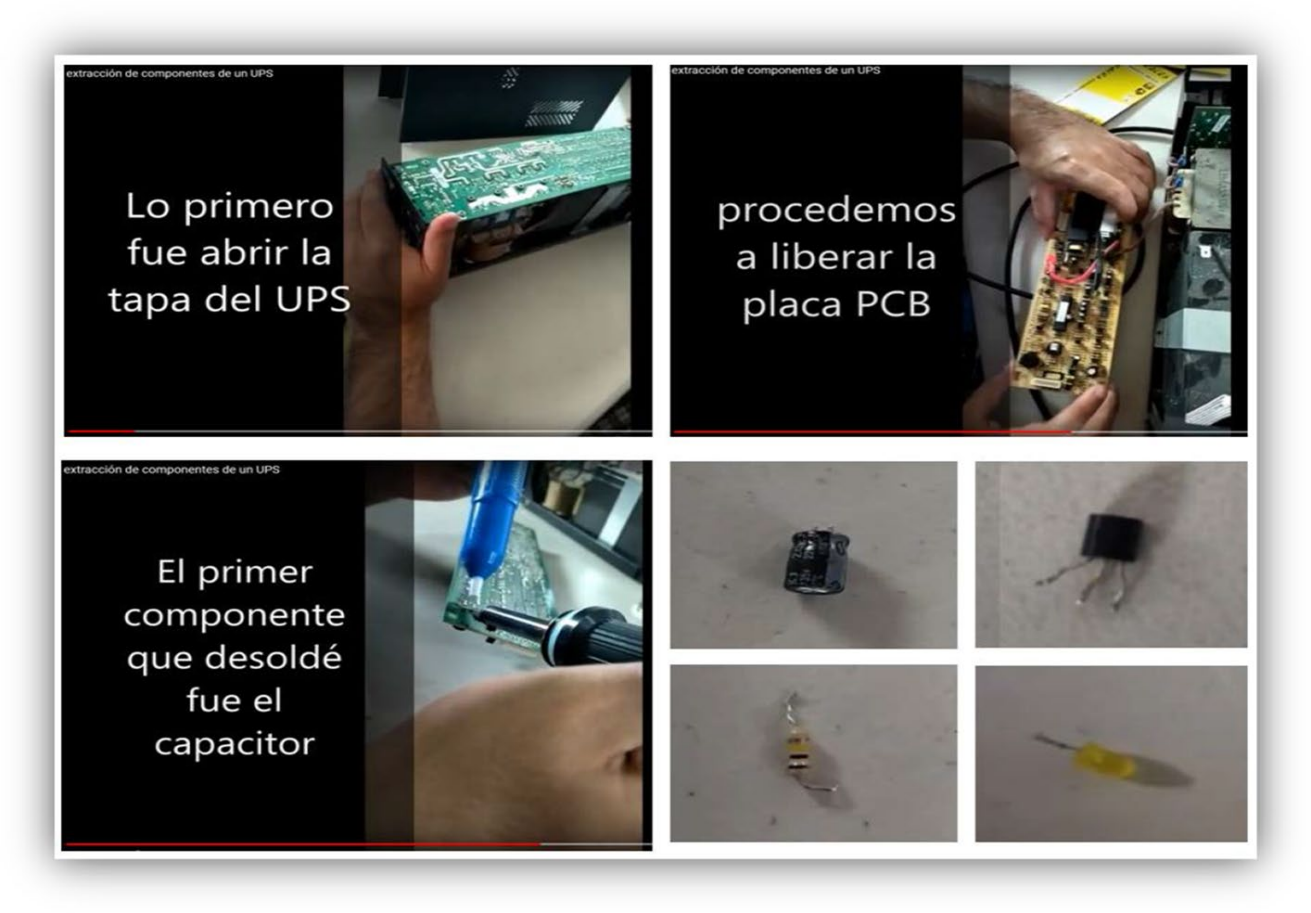
Images of a video of the activity: “Recycling the WEEEs.” The student explains the disassembly of WEEE (UPS) and the extraction process (desoldering) of different usable parts for the construction of the BEAM robot: capacitors, transistors, resistors, and LEDs

The learning and evaluation activities of modules #7 and #8 were focused on BEAM robots and WEEEs. It is important to add that the students were given a UPS discarded from the University to facilitate this activity. All the students disassembled some WEEE and extracted parts, which could be helpful to robots construction. Example images taken from one of the student videos are shown in Fig. 14. In this case, a UPS, and the process of desoldering and extracting parts, such as capacitors, transistors, resistors, and LEDs. This activity developed the ability to recognize the electronic components used in the robot, but now, in a piece of different equipment. In addition, the ability to extract that part and evaluate if it was appropriate for the robot’s construction.

**Fig. 15:**
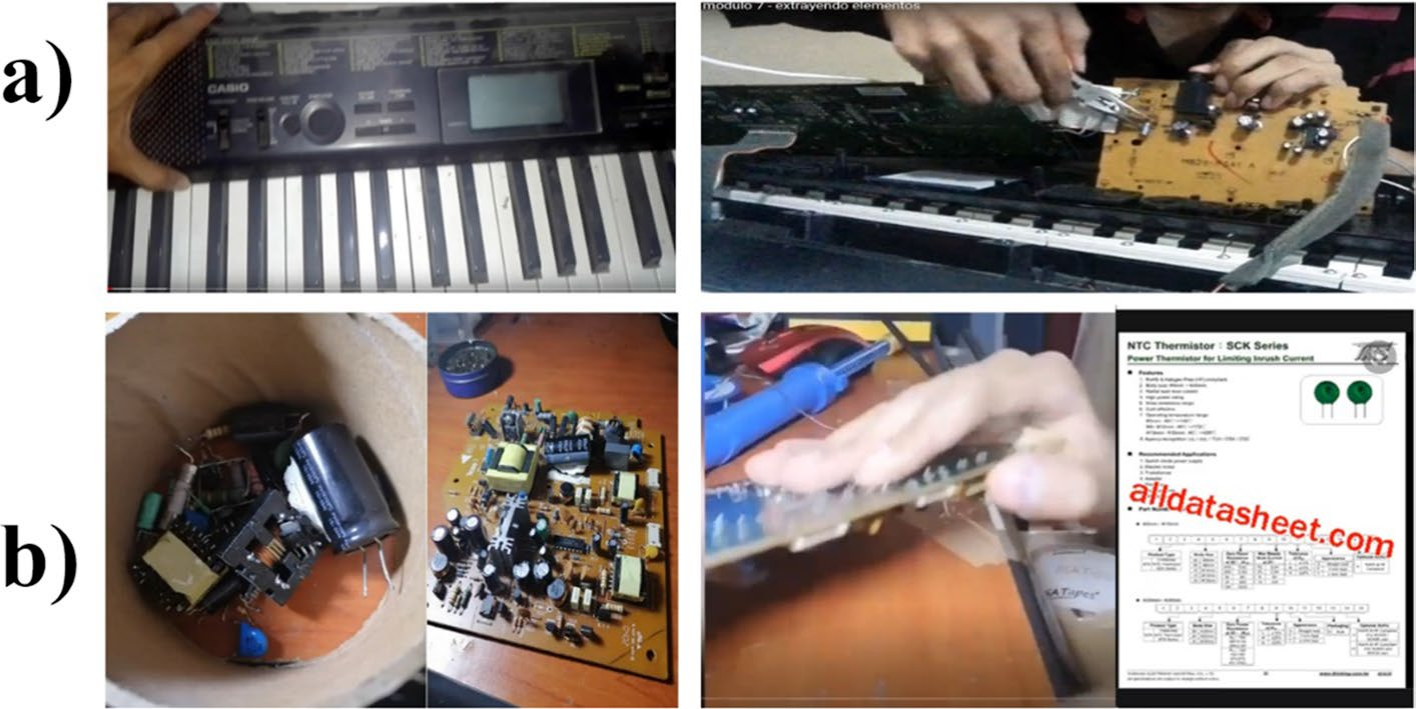
Images of a video of the activity: “Recycling the WEEEs.” In this case, two examples of other WEEEs, apart from the UPS delivered for the activity. **a** An electronic musical instrument. **b** An audio amplifier

It is important to note that five students did not use the delivered UPS; rather, they used other WEEEs in their homes to carry out the recycling activity. Two examples are shown in Fig. 15a an electronic musical instrument and b an audio amplifier. For the latter, the student looked for the datasheet of the extracted parts himself. He commented in his Logbook:

“*To use the transistor in the BEAM robot, it is important to know if it is an NPN or PNP, and you cannot tell just by looking at the transistor. That is why I looked for the code that the transistor has written on the Internet. I learned this in a previous microcontroller course; however, I have never identified such small parts. I shared this experience with my classmates from this course.”*

**Fig. 16:**
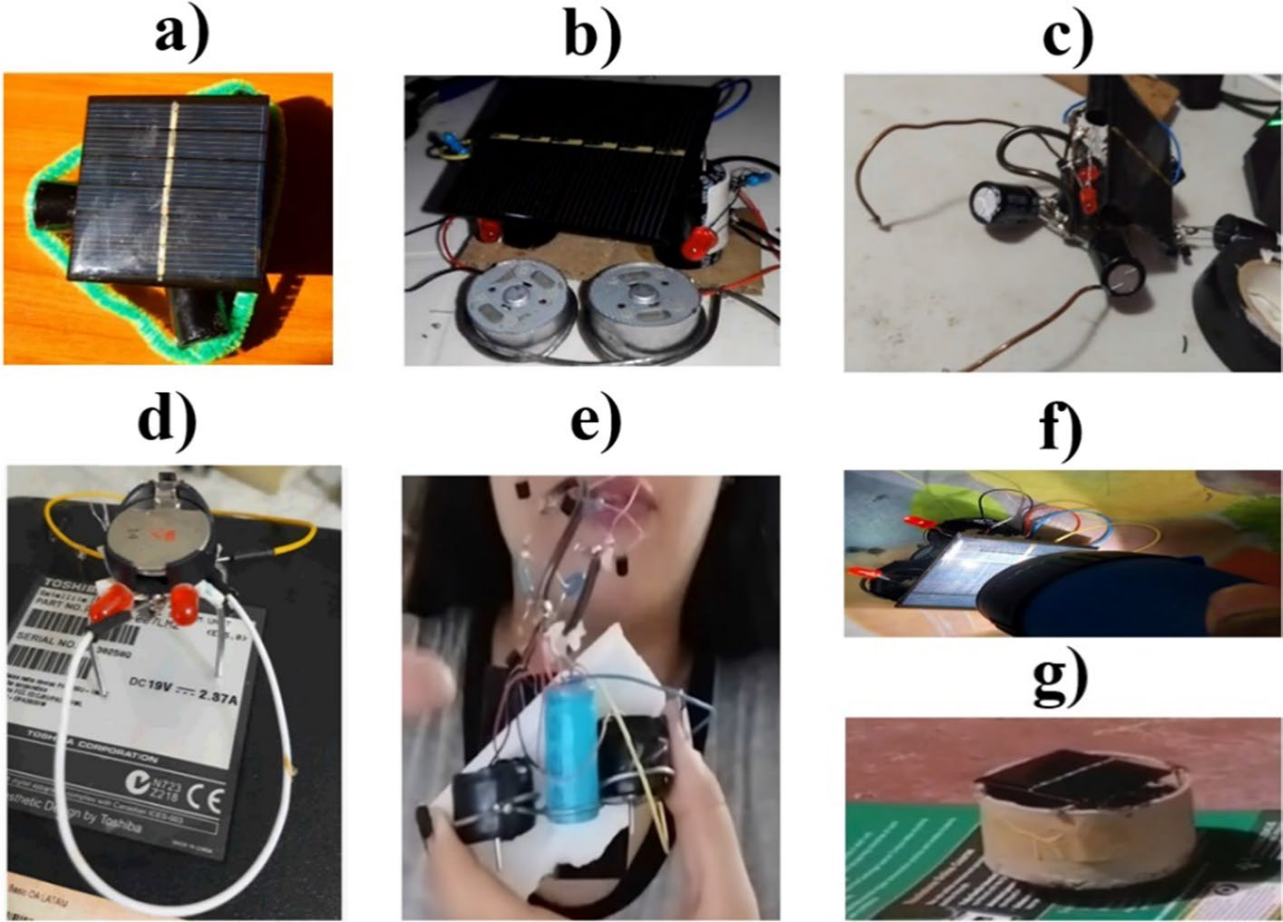
Robots were built in the last module. **a** SYMMET robot with a plastic frame for, instead of metal. **b** PHOTOPOPPER robot with a cardboard structure to integrate all the parts. **c** SYMMET robot with metal parts as an antenna. **d** SYMMET robot ultimately built entirely from recycled parts. **e** and **f** PHOTOPOPPER robots with recycled parts. **g** New design Robot PHOTOPOPPER with a metal cover to protect the electromechanical interior

The last module, #9, deals with creativity and integrating recycled parts into the BEAM robot. The students were challenged to propose a new BEAM robot design, using the parts recovered from the WEEEs. Only seven out of fifteen students completed this activity. Figure 16 shows the seven built robots, in which new morphological structures were created for the robots (Images a–d and g), recovered parts were integrated (images b–d, f and g). Even in one of the robots, a cylindrical plastic piece was used to cover the entire electronic and mechanical structure with an aesthetic vision (Image g).

## Conclusion

The study presented in this work considered whether BEAM robotics and its design, construction, and operation process impacts the development of STEM knowledge and skills focused on the Electrical, Electronic, and Mechanical domains. In addition, Computational Thinking and WEEEs Recycling domains were explored. A course and its respective curriculum were designed and implemented to study this proposal. The course was presented to a sample of fifteen students from the universe of the engineering faculty of the Universidad Interamericana de Panama, and various data collection tools were applied.

A pre and post-survey was applied to the students to evaluate the impact of the course on five dimensions of STEM knowledge and skills: Electrical, Electronics, Mechanics, Computational Thinking, and recycling of WEEEs.

As stated in the survey before the course, the students showed basic knowledge and skills, and after the course, an improvement was evident. It is important to mention that a t-test evidenced a significant change in STEM knowledge and skills in students. However, it should be noted that it cannot be set for all domains, precisely, computational thinking. Better learning and assessment activities may have to be introduced in future courses that can contribute to this knowledge.

In general, the results show that this new teaching tool can promote the STEM curriculum in engineering university students. It is concluded from the learning activities that a tangible constructive process helps to improve the cognitive processes of topics that can be so abstract, such as current and electrical voltage and the functioning of a neural network. Furthermore, although the learning and evaluation activities on computational thinking were not so effective, the students learned to recognize each part of the robots and how these are related; to abstract from an electronic scheme, concretize it and systematize its behavior. Also, thanks to an evaluation of the robot's behavior, they learned to identify the faults and apply the solutions, as in the debugging process carried out in the programming of software.

These results are promising and motivate extending the tool's application and the curriculum to other academic levels, such as secondary and pre-secondary education. As future work, the didactic tool based on BEAM robotics will be evaluated with students who do not have a foundation in the studied domains. In addition, the tool will be extended to other academic levels.

## Data Availability

The datasets used and/or analyzed during the current study are available from the corresponding author on reasonable request.

## Abbreviations

STEM: Science, technology, engineering, mathematics
BEAM: Biology, electronics, aesthetics, mechanics
NV: Nervous network
WEEE: Waste electric and electronic equipment
